# MENA-SINO Consensus Statement on Implementing Care Pathways for Acute Neurovascular Emergencies During the COVID-19 Pandemic

**DOI:** 10.3389/fneur.2020.00928

**Published:** 2020-08-25

**Authors:** Hosam Al-Jehani, Seby John, Syed Irteza Hussain, Amal Al Hashmi, May Adel Alhamid, Dareen Amr, Atilla Ozcan Ozdemir, Ashfaq Shuaib, Adel Alhazzani, Mohammad Ghorbani, Ossama Mansour, Maher Saqqur

**Affiliations:** ^1^Department of Neurosurgery and Interventional Radiology, King Fahad Hospital of the University, Imam Abdulrahman Bin Faisal University, Dammam, Saudi Arabia; ^2^Department of Neurology and Neurosurgery, Montreal Neurological Institute and Hospital, McGill University, Montreal, QC, Canada; ^3^Neurological Institute, Cleveland Clinic Abu Dhabi, Abu Dhabi, United Arab Emirates; ^4^Central Stroke Unit, Ministry of Health of Oman, Khoula Hospital, Muscat, Oman; ^5^Department of Neurology and Interventional Radiology, King Fahad Hospital of the University, Imam Abdulrahman Bin Faisal University, Dammam, Saudi Arabia; ^6^Stroke and Neurointervention Unit, Neurology Department, Alexandria University School of Medicine, Alexandria, Egypt; ^7^Department of Neurology, Neurocritical Care, Eskisehir Osmangazi University, Eskişehir, Turkey; ^8^Department of Neurology, Stroke Center, Eskişehir, Turkey; ^9^Division of Neurology, Department of Medicine, University of Alberta, Edmonton, AB, Canada; ^10^Neurology Division, Department of Medicine, College of Medicine, King Saud University, Riyadh, Saudi Arabia; ^11^Division of Vascular and Endovascular Neurosurgery, Firoozgar Hospital, Iran University of Medical Sciences, Tehran, Iran; ^12^Trillium Hospital, University of Toronto at Mississauga, Mississauga, ON, Canada

**Keywords:** COVID, endovascular therapy, recommendation, MENA, acute ischemic stroke, subarachnoid hemorrhage

## Abstract

In the unprecedented current era of the COVID-19 pandemic, challenges have arisen in the management and interventional care of patients with acute stroke and large vessel occlusion, aneurysmal subarachnoid hemorrhage, and ruptured vascular malformations. There are several challenges facing endovascular therapy for stroke, including shortages of medical staff who may be deployed for COVID-19 coverage or who may have contracted the infection and are thus quarantined, patients avoiding early medical care, a lack of personal protective equipment, delays in door-to-puncture time, anesthesia challenges, and a lack of high-intensity intensive care unit and stroke ward beds. As a leading regional neurovascular organization, the Middle East North Africa Stroke and Interventional Neurotherapies Organization (MENA-SINO) has established a task force composed of medical staff and physicians from different disciplines to establish guiding recommendations for the implementation of acute care pathways for various neurovascular emergencies during the current COVID-19 pandemic. This consensus recommendation was achieved through a series of meetings to finalize the recommendation.

## Background

The novel coronavirus disease (COVID-19) was first identified in the Wuhan province of China in late December 2019 and spread rapidly around the globe. Consequently, a pandemic characterized by a rapid spread through respiratory droplets with human-to-human contact was declared by the World Health Organization on March 11, 2020 (1–4).

### COVID-19 in the Middle East

COVID-19 is the second coronavirus outbreak to affect the Middle East, following the MERS-CoV reported in Saudi Arabia in 2012. The United Arab Emirates (UAE) was the first Middle East country to report a coronavirus-positive case, following the Wuhan coronavirus outbreak in China (4).

### COVID-19 and Stroke

COVID-19 is increasingly being recognized as a cause of thromboembolic phenomena, such as acute ischemic strokes and cerebral venous sinus thrombosis (5).

## Challenges of Intravenous Thrombolysis and Endovascular Therapy (EVT) Interventions

### Patient Avoidance of Seeking Medical Attention

Published and anecdotal reports suggest that during the pandemic, there has been a drastic reduction in the number of stroke patients being evaluated in the emergency room (ER) or being admitted to hospitals worldwide (6). It is highly unlikely that the incidence of stroke has suddenly changed or reduced. This could be explained by the inability to seek medical care due to the extreme restrictions established to limit virus transmission or fear of contracting the virus upon visiting the hospital. In addition tertiary care hospitals may be inundated with COVID-19 patients, and as such, patients with stroke are being treated at secondary-care facilities or may not be transferred to comprehensive stroke centers at all. Clearly, strokes remain an emergency, and patients should seek immediate care despite the current pandemic.

### Healthcare Personnel Shortages

Healthcare personnel have a high risk of becoming infected during this novel pandemic, particularly before transmission dynamics are fully characterized. Of the COVID-19 cases reported to the Centers for Disease Control from February 12 to April 9, a proportion included data on whether the patient was a healthcare worker (HCW) in the USA, with up to 19% of cases identified as healthcare personnel (7). Quarantines for HCWs who have tested positive and for those with high-risk exposures could severely impact the smooth functioning of stroke units and may even jeopardize an entire department if it involves small numbers (e.g., a neurointerventional team). In addition, many hospitals are redeploying clinicians of all specialties to the care of COVID-19 patients, thereby draining resources of care for other medical conditions. Both of the above factors present a substantial challenge, even for well-established stroke centers, and stroke teams will likely experience staff shortages.

### Hospital Beds for Stroke

It is likely that hospitals will be inundated with COVID-19 patients. Specifically, these patients will include individuals who are critically ill from a respiratory viewpoint; these patients will require intensive care unit (ICU) hospitalization, and many will require ventilators. Thus, disruptions in standard protocols such as post-thrombolysis and post-thrombectomy care should be expected, and variations in protocol or abbreviated protocols will be needed to efficiently utilize staffing and bed space while maintaining the best possible patient care. Repatriation from a comprehensive stroke center to lower levels of care following a period of stability after critical procedures, such as mechanical thrombectomy, aneurysm occlusion, or hematoma evacuation, may be reasonable (8).

### Shortage of Personal Protective Equipment

The provision of adequate personal protective equipment (PPE) and clear guidelines on its application are imperative to protect healthcare personnel and to prevent viral spread among HCWs. Given that community transmission of COVID-19 is well-established in most areas, all stroke alerts presenting to the ER should ideally be treated as a potentially infected patient. While the use of PPE for maximum protection, as dictated by international and institutional bodies, is ideal, this practice may not be possible given the PPE shortages that are being encountered in many countries (9). Thus, responses to code stroke may be delayed due to PPE unavailability.

### Anesthesia Challenges

Known or suspected COVID-19 patients as well as carriers will likely require mechanical thrombectomy for large vessel occlusion. This situation poses challenges regarding anesthetic management, given the urgent nature of the procedure and an “unknown” COVID-19 status. COVID-19 has a high risk of spreading through droplets and aerosols (1, 10). Bag-mask ventilation, intubation, extubation, and airway suctioning are aerosol-generating procedures, and any disconnection of the circuit risks further aerosolizing secretions. In theory, monitored anesthesia care (MAC) may prevent intense aerosolization; however, stroke patients undergoing MAC sedation may require supplemental oxygen via a nasal cannula mask or other methods, such as chin-lift or jaw thrust maneuvers, to improve oxygenation, which may increase the degree of airborne exposure to the anesthesia provider and other involved HCWs. Another consideration is the need to convert from MAC to general anesthesia (GA) in a minority of patients. Urgent intubation in a non-negative pressure room introduces an exposure risk to all team members within the room. In addition, workflows with regard to dedicated space for intubation pose additional challenges.

### Delay in Treatment Timelines

While prehospital delays are expected, given travel, and transfer logistics amidst ongoing community lock-downs in many countries, several challenges remain once the patient arrives at the ER. Protocols for protected stroke alerts have been published. Limited neurology personnel, PPE shortages, transfer times through designated corridors/elevators from the ER to imaging with appropriate PPE, unavailability of computerized tomography (CT) scanners during disinfection periods, and time for donning/doffing PPE are all potential factors that may increase door-to-needle times when treating patients with intravenous thrombolysis as well as door-to-puncture for thrombectomy.

### Angiography Suite

To minimize exposure, staffing within the room should be kept to a minimum; however, some patients may be technically challenging, and an additional hand may be extremely helpful. Challenges while operating with multiple layers of PPE are foreseeable. The physical and psychological burden of neurointerventional stroke calls, particularly during the current pandemic, is likely substantial, especially given the small size of these teams. As reported by a recent survey of neurointerventional nurses and radiology technologists from 20 stroke centers in the USA, only 9 centers (45%) had more than 6 nurses or technologists in their call pools for stroke (11). Many institutions with multiple angiography suites are reserving one dedicated suite for suspected COVID-19 strokes or other emergencies.

## MENA-SINO Guiding Recommendations for EVT

As a leading regional neurovascular organization, MENA-SINO has established a task force comprised of physicians, nurses, and medical staff from different disciplines (neurology, neurosurgery, interventional neuroradiology, and neurocritical care) to establish guiding recommendations for the implementation of acute care pathways for various neurovascular emergencies during the current COVID-19 pandemic. These recommendations can be greatly enhanced by telemedicine options to minimize patient–physician interactions, as dictated by clinical needs. Other international entities have also published different guidelines, all aiming at achieving “protected code stroke protocol” (12–14).

We describe the following guiding recommendations to be implemented in the MENA region to facilitate care for patients and to provide optimal protection for HCWs.

**Figure 1:**
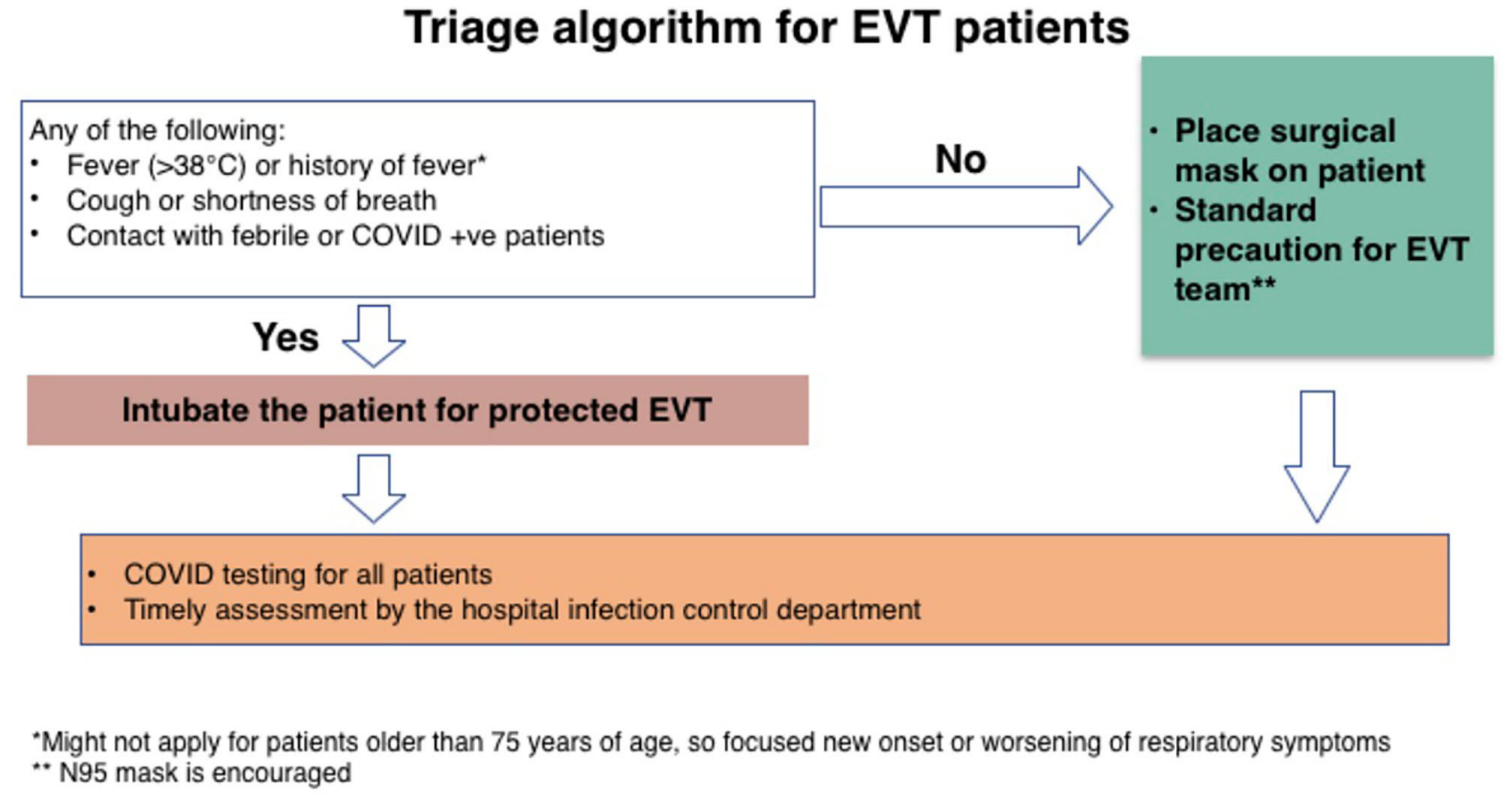
Triage algorithm for EVT patients.

1. Patients must be properly triaged to guide the safety of their clinical encounter (Figure 1).
2. A clear standardized list of priorities for treatment must be established across the different neurovascular pathologies (Table 1). This standardization will render treatments more efficient and will allow for optimal healthcare delivery, by establishing proper operational policies.
3. Outpatient and office visits should be conducted virtually to avoid unnecessary contact between patients and physicians.
4. Each patient should be transferred to another institution if required, as soon as he/she is sufficiently stable to receive the required intervention (Table 1).
5. COVID and non-COVID regions should be designated within the hospital to guide the safety and reciprocity of patient transfers between institutions.

**Table 1:** EVT priority of transfer based on disease entity.

**Category**	**Timeline**	**Disease entity**
Priority 1	Immediate: Acute life- threatening condition or acute transfer within 6 h	Acute ischemic stroke (AIS)
Priority 2	Within 24 h: Loss of life or significant function that can be saved	Coiling or clipping of a ruptured saccular aneurysm with SAH Craniotomy or embolization of a ruptured AVM with prenidal/nidal aneurysms Decompressive craniectomy or hematoma evacuation*
1. If there is not a high risk for in-hospital mortality (inferred from mortality and prognosis assessment scales such as SOFA)		
Priority 3	Within 1 week: Life or significant functional loss that can be saved by intervention within 1 week	Complex ruptured intracranial aneurysm requiring special preparation or equipment
Priority 4	Within 1 month: Life or significant functional loss that can be saved by intervention within 1 month	1. Ruptured AVM with no nidal aneurysms 2. High-grade dural AV fistulae with ICH 3. Carotid revascularization (endarterectomy or stenting) for symptomatic carotid stenosis
Priority 5	After the COVID-19 pandemic	Any other pathology with non-hemorrhagic or ischemic presentation, with a strong recommendation to address risk factors and provide continuous surveillance

The following process is recommended for the referring hospital:

### Prehospital Stage

For all stroke patients presenting directly to the ER or being transferred, the following measures should be taken.

1. Infection control screening: Symptoms or signs of COVID-19, history of infection, contact with infected persons, and travel history should be obtained by emergency medical services (EMS) personnel evaluating the patient at the first encounter.
2. All patients should wear a surgical mask when able, irrespective of the screening outcome.
3. Pre-notification: Hospitals should be pre-notified regarding stroke specifics, COVID screening results, and suspicious cases, if any.
4. When available, telemedicine should be utilized for triage in the prehospital phase. If telemedicine is not available, off-label use of social messaging applications for remote medical consultation can be used, although caution is needed to respect patient data confidentiality.

### Hospital Stage

#### Emergency Room

1. The ER should be divided such that separate spaces and corridors are available for COVID and non-COVID patients.
2. To screen for symptoms of COVID-19 in expedited manner to conform to the time-critical nature of stroke care (Figure 1).
3. All patients should wear a surgical mask.
4. Patients with a positive infection screen or those highly suspected, as reported by emergency medical services (EMS) or ER personnel should be roomed in a dedicated COVID area or negative-pressure room if available.
5. Telemedicine (telestroke) should be utilized when available to obtain history and to perform a neurological examination in order to limit direct contact between medical staff and patients.
6. A protective code stroke protocol should be established for patients with a positive COVID screen. One member of the stroke team should perform the evaluation and provide therapy donning full PPEs.
7. A scoring system should be established for risk stratification of HCW exposure and risk of COIVD-19 infection (Table 2).

**Table 2:** Healthcare worker exposure risk stratification.

**Risk attribute for contact with COVID-19 patient**	**Score 1**	**Contact duration**	**Score 2**	**Distance**	**Score 3**	**Total score**	**Risk stratification**
Health worker wearing proper protective gear	0	Less than 20 min	1	More than 1.5 m (6 feet)	1	_________	Score 2–3: Low risk
		More than 20 min	2	Less than 1.5 m (6 feet)	2		
Health worker NOT wearing proper protective gear	1	Less than 20 min	1	More than 1.5 m (6 feet)	1		Score 4–5: High risk
		More than 20 min	2	Less than 1.5 m (6 feet)	2		

*Adopted from the MOH, Saudi Arabia*.

#### Acute Imaging

1. Standard hospital imaging protocols should be followed for acute stroke treatments.
2. If multiple CT rooms are available, a dedicated CT scan room for COVID-19 patients should be established, provided that the addition of the chest CT does not incur a treatment delay of more than 5 min.
3. If positive pulmonary symptoms are present, consider performing low-dose chest CT simultaneously with head CT using head and neck CT angiography (CTA). It is also wise to include chest CT in the stroke protocol, as PCR tests and the absence of pulmonary symptoms do not exclude the possibility of COVID-19 infection.
4. Avoid multiple visits to the CT room to minimize exposure.
5. For acute imaging, magnetic resonance imaging (MRI) should be avoided if possible. The use of MRI should be restricted to absolute necessities, following the guidance provided by the American College of Radiology on the use of MRI (15).

### Acute Stroke Treatment: Intravenous Thrombolysis and Endovascular Thrombectomy

1. Patients should continue to be treated according to current standard guidelines for intravenous thrombolysis and endovascular mechanical thrombectomy, with the best adherence possible (16).
2. Although there are some situations for which the current guidelines have no clear answers or strong recommendations, the decision to treat a patient should take into account the seriousness of the COVID-19 disease and prognosis, particularly with regard to endovascular thrombectomy. For patients with evidence of multi-organ dysfunction or critical illness, outcomes of endovascular intervention may be suboptimal, and the risks and benefits of such procedures must be weighed against the consumption of resources and potential exposure to caregivers. A multidisciplinary discussion among the treating physicians should be held to make the most appropriate treatment decision.

### Airway Management

1. The anesthesia team should be notified as soon as possible regarding potential endovascular procedures.
2. Conscious sedation should be considered as first-line treatment for patients with acute stroke interventions, if the patient is stable. However, a low threshold should be maintained for intubation in patients with respiratory distress, inability to protect the airway, posterior circulation stroke, vomiting, or agitation and for those who are uncooperative.
3. Intubation should be performed in a negative-pressure room separate from the angiography suite or in a dedicated angiography suite with negative pressure capabilities. In the absence of negative pressure, an aerosol box can be a good substitute in special situations (17).
4. Extubation should be avoided in the angiography suite and instead be performed in a negative-pressure room.
5. Transfers should avoid breaking the initial ventilator circuit, bagging, or reconnection to a new ventilator.

The following process is recommended for the referring hospital:

1. Acute ischemic stroke
  - All patients must be screened for COVID-19. The use of telecommunication is recommended if available.
  - Any patient with fever or respiratory symptoms should be disclosed upon the referral request to ensure proper precautions [as a COVID-19-positive individual or person under investigation [PUI]] and to ensure that trends of vital signs are properly recorded.
  - The National institute of health stroke scale (NIHSS) must be documented, and the score must exceed 5 for a transfer to be considered.
  - Perform brain non-contrast CT (NCCT) to rule out hemorrhage or the presence of an established infarction (Alberta stroke program early CT score (ASPECTS) above 6).
  - Perform CTA to confirm the occurrence of large vessel occlusion and CT perfusion to identify a mismatch. A higher large vessel occlusion (LVO) score may be acceptable for transfer, depending on local logistic preparedness.
  - Patients with a high risk of COVID-19 should wear a surgical mask.
  - Obtain chest CT at the time of the initial CT (ground-glass appearance).
  - If the patient presents with an unknown time of onset or a delayed onset, the option of obtaining a CT perfusion or a brain MRI should be offered in the receiving hospital to assess any mismatch prior to a consideration of transfer. If this procedure is not feasible, the ASPECTS should guide the transfer, with tissue imaging performed in the receiving stroke center.
    ^**^ Intravenous tissue plasminogen activator (IV-tPA) eligible patients should receive thrombolysis based on the protocol in the referring hospital (telestroke managed or guided by stroke neurology).
2. Aneurysmal subarachnoid hemorrhage (aSAH)
  - All patients must be screened for COVID-19, as described above.
  - Any patient with fever or respiratory symptoms should be disclosed upon the referral request to ensure proper precautions (as a COVID-19-positive individual or PUI) and to ensure that the trends of vital signs are properly recorded.
  - Glasgow coma scale (GCS) and World federation of neurosurgical societies (WFNS) grades must be documented (consideration for transfer: GCS > 9 and WFNS grade 1–3).
  - Perform brain NCCT to document the SAH and to exclude intraventricular hemorrhage or intracerebral hemorrhage (IVH-ICH) and hydrocephalus requiring an external ventricular drain (EVD).
  - Perform CTA to confirm the presence of intracranial aneurysm and to rule out any other vascular pathology.
  - Obtain chest CT at the time of the initial CT (ground-glass appearance) for the above-mentioned reasons.
  - If the CTA is negative for aneurysm, the transfer should be aborted, and repeat vascular imaging (CTA or digital subtraction angiography [DSA]) should obtained within 7 days.
    ^**^ In the case of a high-grade SAH, improvement after the EVD insertion should warrant a referral request.
3. After the need for an interventional procedure has been confirmed, the following steps should be taken:
  - The patient should be intubated in the referring hospital, and a closed circuit should be ensured throughout the process of transferring from and to the referring hospital.
  - The patient must be accompanied by a medical transfer team that follows strict PPE precautions. This team should be equipped to offer hemodynamic and ventilatory support during the transfer.
  - The patient should be connected to a portable ventilator that will be used throughout the transfer process, including the angiographic procedure, to ensure that the closed ventilatory circuit is not interrupted.

Precautions during the endovascular procedure

All patients should be treated as though there is a high suspicion of the patient being COVID-19-positive. Accordingly, the following precautions should be implemented:

1. PPE for all staff coming in contact with the patient
  - Lead apron and a yellow gown
  - Head cover
  - N-95 mask covered by a regular mask
  - Goggles
  - Face shield
  - Sterile gown and gloves
2. Strict and supervised movement between different zones in the angiography suite
  - Cold zone outside the angiography suite (green zone: control room)
  - Intermediate zone (yellow zone: scrubbing area)
  - Hot zone (red zone: inside the angiography suite)
3. The angiogram set, pressure bags, and basic access catheters should be prepared for use before the patient enters the angiogram suite. Ready-to-use verapamil and heparin syringes should be included in the angiogram set, and a vial of actylase (IV tPA) should be available in the cold zone. Other antiplatelet agents can be added based on local protocols.
4. A high-efficiency particulate air (HEPA) filter should be placed by the door of the angiogram suite.
5. All closets and cabinets should be closed during the angiography procedure.
6. The room should be labeled as COVID-19-POSITIVE, with no entry other than the angiography team.
7. Intervention technicians should remain in the cold zone to limit exposure and to facilitate material handling.
8. The circulating nurse should remain in the cold zone, supervising the movement of personnel and the donning/doffing of PPE upon entry and exit of any angiography team member.
9. The angiography team in the hot zone should consist of
  - One scrub nurse
  - Up to two interventionists
  - One anesthesia physician, with strict control on the airway to avoid suctioning and aerosol leaks
10. All interventional material should remain outside the angiography suite (in the cold zone) and should be handed to the scrub nurse upon request.
11. The arterial access sheath should be removed at the end of the angiographic procedure. Manual compression or a closure device should be utilized for hemostasis.

^**^ Depending on the policy implemented, the patient can either remain in the treating hospital or be transferred back to their referring hospital.

F. Teamwork during the pandemic
  F.1. In any given region, neurovascular centers are advised to reduce the number of healthcare staff on clinical duty during the pandemic. We emphasize that all HCWs should continue to take universal precautions and utilize PPE as guided by the Ministries of Health and/or local institutional infection control protocols.
  F.2. Cross-privileging of neurointerventionalists during the pandemic should be implemented if the need arises. This approach will allow for continuous care of patients needing these interventions.

### Psychological Support for Healthcare Workers

A recent study assessing the magnitude of mental health outcomes and associated factors among HCWs treating patients exposed to COVID-19 in China reported symptoms of depression (634; 50.4%), anxiety (560; 44.6%), insomnia (427; 34.0%), and distress (899; 71.5%) (18).

Healthcare providers may benefit from following the measures listed below.

▸ Self-monitor and pace.
▸ Regularly check in with colleagues, family, and friends (check-ins may need to be virtual).
▸ Take brief relaxation/stress management breaks.
▸ Establish a COVID-free discussion zone.
▸ Seek reliable information and proper expert assessments to assist in making informed decisions if needed.
▸ Focus efforts on what is within your power.
▸ Check in with other colleagues to discuss work experiences.
▸ Provide consultations and collegial support (remotely).
▸ Allow for “hot debriefs,” e.g., following the STOP-5 approach (Summarize, Things that went well, Opportunities to improve, Point to action and responsibility) adapted from the Edinburgh emergency medicine model developed by (19).
▸ Schedule time off from work for gradual reintegration into personal life.
▸ Prepare for worldview changes in one's life that may not be mirrored by others.

## Closing Remarks

Despite the current challenges encountered in the EVT treatment of acute stroke and neurointervention, there remain opportunities to learn from the current pandemic experience, with applications for future disasters.

In summary, our main recommendations are the following:

1- The risk of COVID-19 (Figure 1) should be stratified in order to prioritize and optimize (Table 1) the utilization of available resources during the COVID-19 pandemic in the MENA region, where resources are limited.
2- The role of telestroke in acute and clinical settings is critical for avoiding unnecessary contact between patients and physicians during the pandemic and to better utilize specialized stroke physicians with limited resources.
3- In an acute ischemic stroke, stringent prescreening criteria should be implemented to distinguish high-risk COVID-19 patients from low-risk patients, with subsequent stroke protocols based on the COVID-19 risk.
4- In aSAH patients, prescreening should be performed before admission and intervention, and a stringent high-risk protocol should be followed in the Neuro-ICU and during intervention based on prescreening results.

In conclusion, the MENA-SINO statement provides guidance to interventionalists and hospitals for prioritizing medical care for neurovascular patients. While these guidelines consider patient safety and infection protective protocols, they do not replace sound clinical judgment, the consideration of patient-specific factors, or institutional policies and procedures.

## Author Contributions

HA-J and SJ: concept design, writing of the manuscript, provision of protocol sections, and revision of the final document. OM, MS, DA, and MA: writing of the manuscript, provision of protocol sections, and revision of the final document. AO, AS, AA, MG, and AAH: provision of protocol sections and revision of the final document. SH: writing of the manuscript and provision of protocol sections. All authors contributed to the article and approved the submitted version.

## Conflict of Interest

The authors declare that the research was conducted in the absence of any commercial or financial relationships that could be construed as a potential conflict of interest.
